# Platypnea-Orthodeoxia Syndrome Revealing an Undiagnosed Patent Foramen Ovale: A Case Report

**DOI:** 10.7759/cureus.100003

**Published:** 2025-12-24

**Authors:** Rachelle Makhoul, Ahmad H Alhajj Mohammad, Nada Saleh, Dina Badran, Tony E Bechara

**Affiliations:** 1 Cardiology, Lebanese University Faculty of Medicine, Beirut, LBN; 2 Internal Medicine, Lebanese University Faculty of Medicine, Beirut, LBN; 3 Cardiology, Lebanese Hospital Geitawi, University Medical Center, Beirut, LBN

**Keywords:** adult congenital heart disease (achd), heart septal defects, internal medicine-cardiology, interventional and structural cardiology, intracardiac shunt, patent foramen ovale, platypnea-orthodeoxia syndrome, transcatheter closure device, transcatheter repair, upright hypoxemia

## Abstract

Platypnea-orthodeoxia syndrome (POS) is an uncommon clinical entity characterized by position-dependent hypoxemia that worsens when standing and improves when lying down. Patent foramen ovale (PFO) was the most commonly reported site of an intracardiac shunt. In addition to PFO, intracardiac shunt leading to POS has been reported from either an atrial septal defect (ASD) or an atrial septal aneurysm (ASA). In cases where POS occurs due to intracardiac shunting in the absence of pulmonary hypertension, closure of the defect can be curative.

We report a rare case of POS caused by a PFO in a 76-year-old woman. Transesophageal echocardiography was essential for identifying the shunt, excluding the presence of pulmonary hypertension, enabling successful percutaneous closure and complete resolution of her symptoms. This case adds novelty beyond typical PFO-related POS due to delayed diagnosis despite prior thromboembolic events.

## Introduction

Platypnea-orthodeoxia syndrome (POS) is an uncommon clinical entity characterized by positional dyspnea (platypnea) and arterial desaturation (orthodeoxia), which worsen in the upright position (sitting or standing) and improve when supine [1].

The first case of POS was reported by Burchell et al. in a patient in 1949 with a post-traumatic intrathoracic arteriovenous shunt [2]. The terms ‘platypnea’ and ‘orthodeoxia’ were coined by Altman et al. and Robin et al. in 1969 and 1976, respectively. In these patients, POS was a result of hepatic and pulmonary disease, respectively [3,4]. In 1984, POS was first described in a patient with an intracardiac right-to-left shunt with orthostatic accentuation of hypoxemia in the absence of hepato-pulmonary dysfunction or elevated right heart pressures [5].

The diagnostic criteria for POS include a decrease in arterial oxygen tension (PaO_2_) of more than 4 mmHg, accompanied by a reduction in oxygen saturation (SaO_2_) of over 5% upon transitioning from a supine to an upright position [6].

Although the underlying mechanisms of POS remain incompletely understood, they are broadly categorized into intracardiac and extracardiac causes [7]. The most frequently implicated intracardiac etiology is a patent foramen ovale (PFO) [8], which permits a right-to-left shunt in the absence of significant pulmonary hypertension [9]. This shunt may also lead to paradoxical embolism, manifesting as cryptogenic stroke, myocardial infarction, or visceral and peripheral ischemia [9].

To note that the presence of anatomical or mechanical factors like ascending aorta dilatation and atrial septal distortion can further predispose to POS in elderly patients due to increased shunting.

Agitated saline contrast echocardiography detects right-to-left shunting across a PFO. Contrast is injected during Valsalva strain and imaged on release, with optimal visualisation at an angle of ~90°, reflecting the typical cranial orientation of the fossa ovalis [10].

We present a case of POS secondary to a PFO in the absence of pulmonary hypertension, who had a history of paradoxical embolism that went uninvestigated, which raised our suspicion of PFO. Our case emphasizes the diagnostic complexity, highlights the utility of echocardiographic assessment with the use of agitated saline, and demonstrates a favorable clinical outcome following definitive intervention with closure device placement [11].

## Case presentation

A 76-year-old woman, a non-smoker, with a known allergy to ibuprofen and a past medical history that is significant for hypertension, type 2 diabetes mellitus, and dyslipidemia. Sixteen years ago, she developed a deep vein thrombosis (DVT) following a lumbar discectomy and instrumentation for a herniated lumbar disc. The postoperative period was complicated by prolonged immobilization, during which the patient remained bedridden for several days. Two weeks after the surgery, she developed a pulmonary embolism (PE), followed one week later by an ischemic stroke involving the thalamus. The stroke was complicated by post-thalamic syndrome and residual left-sided weakness. Notably, both the pulmonary embolism and ischemic stroke were not formally investigated at the time. Since then, the patient has been maintained on warfarin 2 mg once daily as a therapeutic anticoagulation.

The patient presented to the Emergency Department with dyspnea and oxygen desaturation (SaO_2_ of 87% on room air). She denied fever, cough, chest pain, heartburn, headache, visual disturbances, rhinorrhea, nausea, vomiting, or diarrhea. One week prior, she had been hospitalized at another facility for pneumonia, treated as an inpatient for five days, and discharged on oral levofloxacin. According to her daughter, the patient was stable at home until an incidental pulse oximetry reading showed SaO_2_ of 82% while sitting.

On presentation, she was alert, oriented, and conscious. On examination, the patient was afebrile with a temperature of 36.8°C, a heart rate of 75 beats per minute, and a blood pressure of 123/75 mmHg. The respiratory rate was elevated at 28 breaths per minute, with an oxygen saturation of 87% on room air while the head of the bed was elevated. Physical examination revealed central cyanosis without finger clubbing, lower limb edema, distended neck veins, or signs of DVT. Chest examination showed normal bilateral air entry with no added sounds. Cardiac evaluation was unremarkable, with normal S1 and S2 heart sounds and no audible murmurs. The abdominal examination revealed no significant abnormalities, and electrocardiography (ECG) demonstrated a normal sinus rhythm.

On initial assessment, the patient was dyspneic with an oxygen saturation (SaO_2_) of 87%, which improved with supplemental oxygen therapy. Arterial blood gas analysis revealed findings consistent with hypoxemia, with a partial pressure of oxygen (PaO_2_) of 48 mmHg. There were no signs of systemic infection with negative inflammatory markers, including C-reactive protein (CRP) and procalcitonin, and the D-dimer level was within normal limits. Relevant laboratory data are summarized in Table 1.

**Table 1:** Laboratory test results. ALT: alanine aminotransferase; GGT: gamma-glutamyl transferase; BNP: brain natriuretic peptide.

Test	Result	Reference range
Complete blood count		
White blood cell count	10.07 × 10^9/L	4.0-11.0 × 10^9/L
Hemoglobin	15.8 g/dL	13.5-17.5 g/dL
Hematocrit	48.2%	41-53%
Platelet count	249 × 10^9/L	150-400 × 10^9/L
Neutrophils	65%	40-70%
Lymphocytes	24%	20-45%
Biochemistry panel		
Creatinine	0.97 mg/dL	0.6-1.3 mg/dL
ALT	29 U/L	7-56 U/L
GGT	52 U/L	9-48 U/L
Bilirubin (total)	0.4 mg/dL	0.1-1.2 mg/dL
Bilirubin (direct)	0.2 mg/dL	0.0-0.3 mg/dL
Cardiac markers		
Pro-BNP	96.11 pg/mL	<125 pg/mL
Troponin	55.53 ng/L	<14 ng/L
Inflammatory markers		
C-reactive protein	1 mg/L	<5 mg/L
Procalcitonin	0.06 ng/mL	<0.1 ng/mL
Coagulation markers		
D-dimer	<125 ng/mL	<500 ng/mL
Screening tests		
SARS-CoV-2 nucleocapsid antigen	Negative	Negative
Influenza A & B rapid test	Negative	Negative

**Figure 1:**
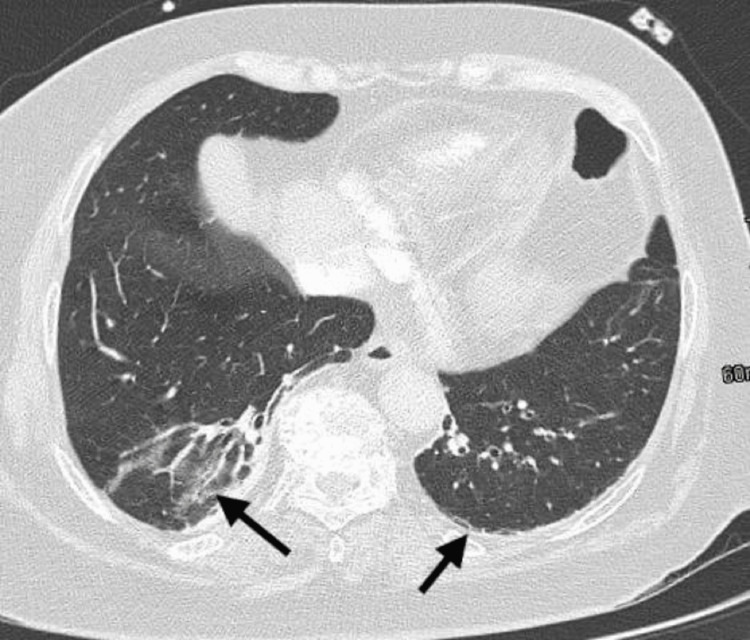
CT angiography of the chest showing bilateral atelectatic changes of the lower lobes (right >>left). The rest of the lung parenchyma is clear; no masses or consolidations, and no suspicious nodules. CT: computed tomography.

**Figure 2:**
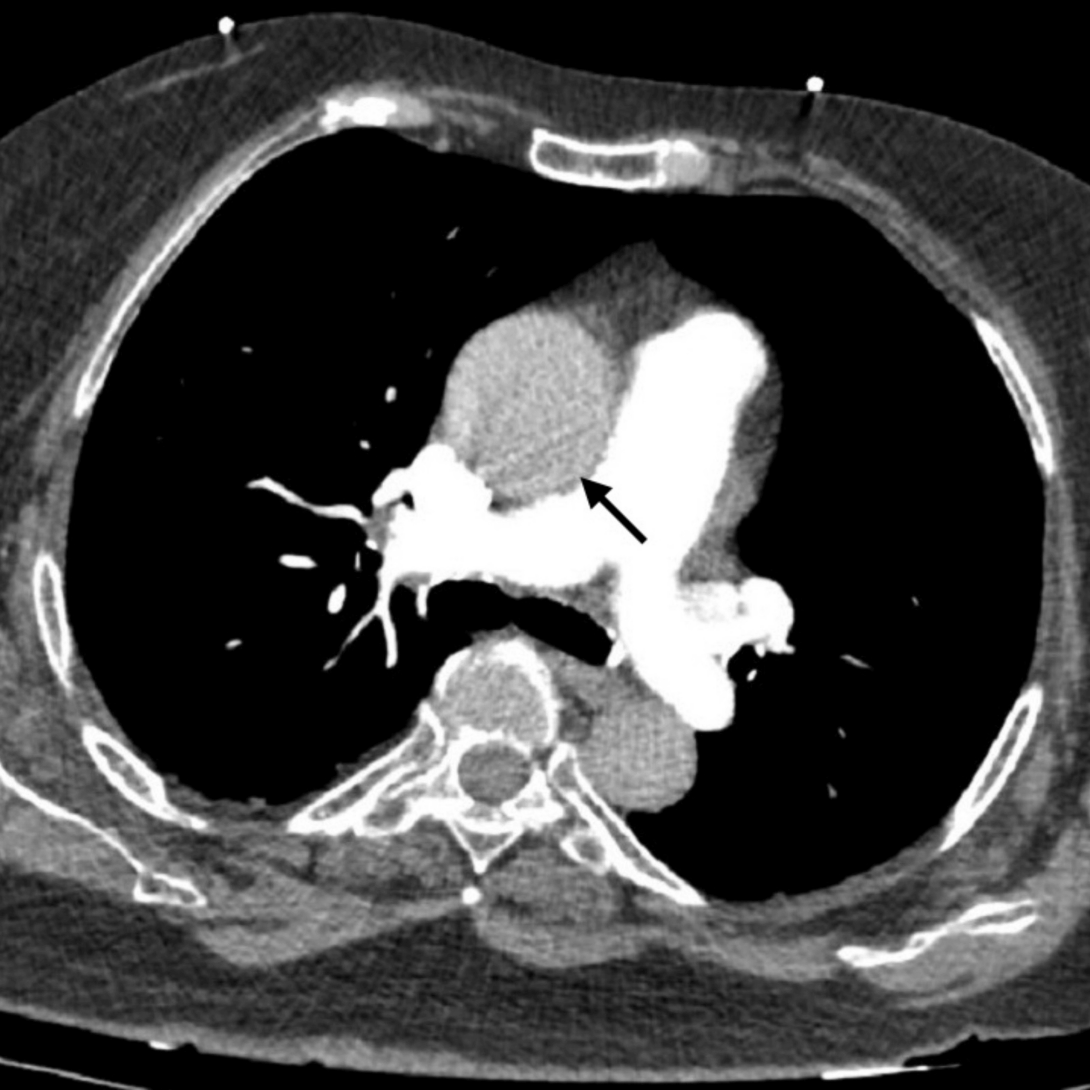
CT angiography of the chest showing mild dilatation of the ascending thoracic aorta (43 mm), as well as no filling defect in the major branches of pulmonary trunk. CT: computed tomography.

Chest X-ray (CXR) showed no infiltrates or consolidation, presence of bi-basilar thin atelectatic bands, and no evidence of pleural effusion. CT angiography of the chest excluded the presence of pulmonary embolism. It revealed atelectatic changes in the lower lobes (Figure 1), an enlarged ascending thoracic aorta measuring 43 mm was also noted (Figure 2), and was attributed to hypertension, atherosclerosis, and the patient's age.

Respiratory status improved with high-flow nasal cannula; however, the patient exhibited marked desaturation (from 95% to 70%) and central cyanosis when assuming an upright position, which improved significantly when lying supine, suggestive of positional hypoxemia. Arterial blood gas (ABG) was not obtained during this episode due to the patient's poor clinical tolerance.

The patient was admitted to the intensive care unit (ICU) for further evaluation, and warfarin was shifted to enoxaparin. Non-contrast-enhanced trans-thoracic echocardiography (TTE) showed a normal left ventricle (LV) size and function, no significant valve diseases, mild tricuspid regurgitation with normal RV function, and non-elevated pulmonary pressures (echo-guided estimated systolic pulmonary artery pressure is 18 mmHg). Subsequently, a trans-esophageal echocardiogram (TEE) with color Doppler and a bubble study using agitated saline was conducted, since transthoracic echocardiographic (TTE) findings were inconclusive. This revealed a large PFO with mixed right-to-left and left-to-right shunt on color Doppler (Video 1).

**Video 1:** TEE showing a large PFO with mixed right-to-left and left-to-right shunt on color Doppler. TEE: transesophageal echocardiography; PFO: patent foramen ovale.

**Figure 3:**
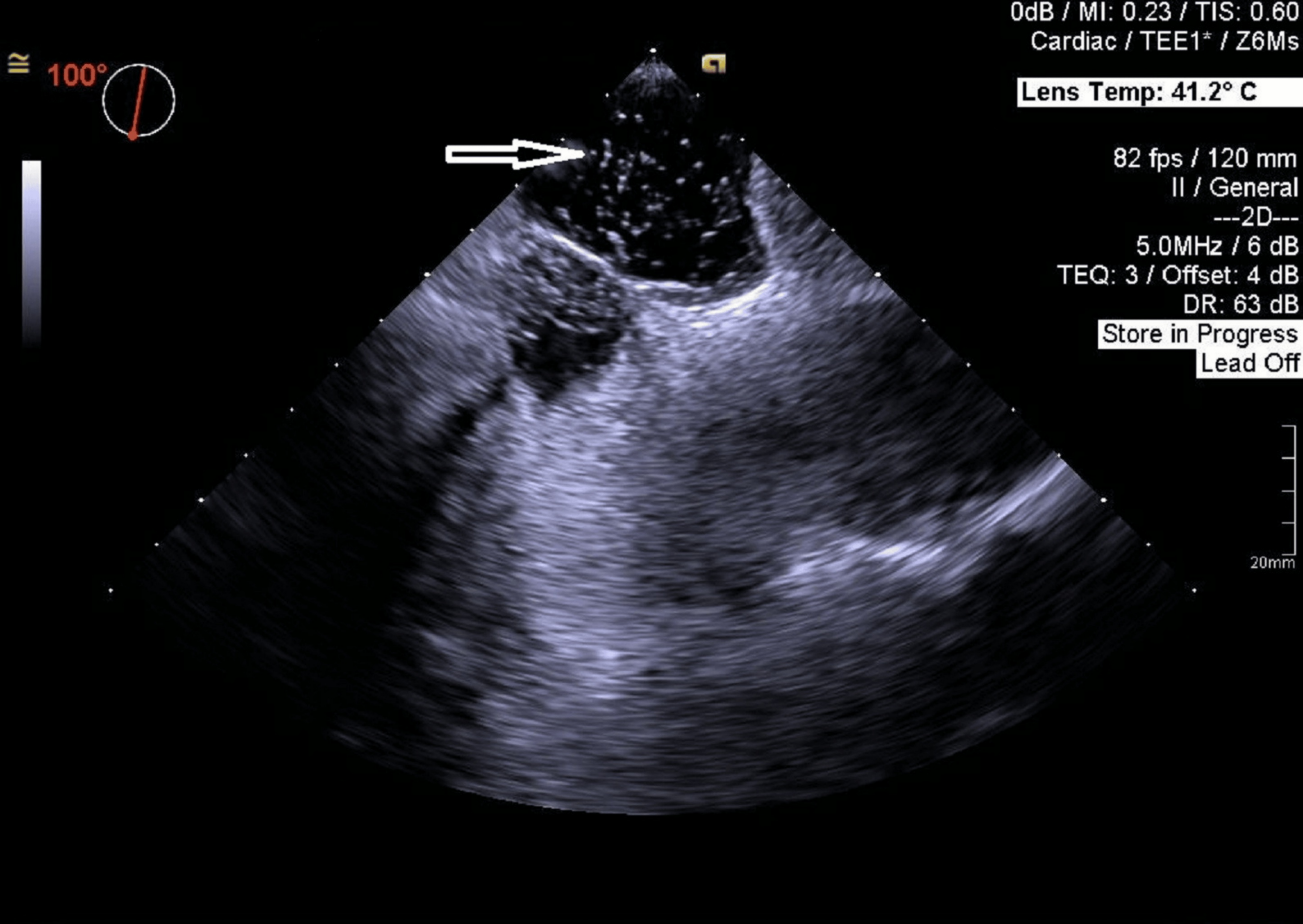
TEE showing bubbles crossing the PFO after two cardiac cycles. TEE: transesophageal echocardiography; PFO: patent foramen ovale.

Bubble study is positive, with bubbles crossing from the right atrium after two cardiac cycles without Valsalva (Figure 3, Video 2).

**Video 2:** TEE with a positive bubble study; bubbles crossing from the right atrium after two cardiac cycles without Valsalva. TEE: transesophageal echocardiography.

These findings are consistent with a diagnosis of platypnea-orthodeoxia syndrome secondary to a large PFO. The presence of mild ascending aorta dilatation further predisposed to POS in our elderly patients due to increased shunting.

Three days later, the patient underwent percutaneous closure of the patent foramen ovale (PFO). A 30-mm AMPLATZER™ PFO Occluder (Abbott Laboratories, Abbott Park, IL, USA) device, mounted on a 0.035-inch guidewire and delivered through a 6 French main pulmonary artery (MPA) catheter, was selected for the procedure (Video 3).

**Video 3:** A 30-mm AMPLATZER™️PFO Occluder device placement was performed under TEE guidance, with no intraprocedural complications. PFO: patent foramen ovale; TEE: transesophageal echocardiography.

Device placement was performed under transesophageal echocardiographic guidance, with no intraprocedural complications observed. Correct positioning of the device was confirmed using both transesophageal echocardiography and a back-and-forth maneuver.

Post-procedure, there was no evidence of residual shunting. The patient's platypnea-orthodeoxia syndrome (POS) symptoms significantly improved. Oxygen saturation increased to 95% in the upright position and 96% in the supine position, as measured by pulse oximetry. The patient was discharged on apixaban 5mg twice daily in stable condition. The patient was symptom-free in a follow-up visit in the clinic four weeks later, with normal oxygen saturation in both upright and supine positions.

## Discussion

Patent foramen ovale (PFO) is common in the general population, with an estimated incidence at 25%-30% [12]. Platypnea‐orthodeoxia syndrome (POS) is a rare and underdiagnosed clinical entity that requires a high index of suspicion because symptoms can mimic other causes of hypoxemia, such as pulmonary embolism, chronic lung disease, or heart failure [13]. POS causes can be due to intracardiac or extracardiac shunts [14]. Approximately 87% of cases are due to intra-cardiac shunting by a PFO (67% of all shunt types), followed by atrial septal defect (ASD) (12%), fenestrated atrial septal aneurysm (ASA), partial anomalous pulmonary venous return, transposition of the great vessels, and unroofed coronary sinus [6,15,16]. Extra-cardiac causes can be grouped into intrapulmonary shunts, ventilation-perfusion mismatch, pulmonary arterio-venous malformations, or hepato-pulmonary syndrome [17,18], all of which were excluded in our patient. In patients with hepatopulmonary syndrome leading to POS, liver transplantation is the only definitive therapy. After transplantation, 80% patients showed improvement in oxygenation. No other medical treatment has shown effective results.

The pathogenesis of hypoxemia in POS related to PFO can be hemodynamic and anatomical [19]. It is thought to be linked to a rise in right atrial pressure or alterations in the extent of right-to-left shunting with upright posture due to underlying structural abnormalities (such as aortic root dilatation, changes in thoracic anatomy) or changes in intra-thoracic or intra‑abdominal pressures (surgery, lung disease, pneumonia), or even mild lung disease [15]. In many cases, pulmonary hypertension is absent [20]. Other extracardiac causes of POS were also excluded. Since it was first described in 1949, more cases have gradually been identified with attempts to explore possible triggers for POS in the absence of an elevated right‐to‐left pressure gradient [5]. Most of the available literature consists of individual case reports, though a few small case series have involved patients with significant lung diseases.

TTE with contrast echocardiography can be used for detecting a PFO, but transesophageal echocardiogram (TEE) with a bubble study is the gold standard tool due to its superior spatial resolution and ability to visualize posterior cardiac structures, including the interatrial septum [21]. Transcranial Doppler (TCD) is an alternative method for detecting PFO after intravenous injection of saline contrast medium and is sometimes considered to be superior to the use of two-dimensional echocardiography [22]. PFO closure (percutaneous and more rarely surgical) is by far the most frequently guideline-supported treatment to alleviate symptoms and correct hypoxemia [11]. Several studies report that percutaneous closure leads to rapid and sustained symptomatic relief, with marked improvements in arterial oxygen saturation and quality of life within days of the procedure [23].

In our case, the standard diagnostic paradigm was followed by recognizing positional hypoxia, ruling out pulmonary hypertension or major lung disease by CT imaging, and documenting a PFO shunting via TEE, along with a mildly dilated ascending aorta.

The decision to proceed with percutaneous PFO closure (AMPLATZER™ PFO Occluder device) aligns with the best available evidence for cardiac POS [11]. Our patient experienced a rapid improvement in oxygenation and symptoms, similar to cases such as those in the Blanche et al. series, where all patients who underwent device closure improved in New York Heart Association (NYHA) class and oxygen saturations [17]. A transthoracic echocardiogram (TTE) was performed one month after device placement. It demonstrated the absence of residual shunting, and the patient reported no symptoms during this follow-up period.

Our case adds to the growing body of evidence that PFO‑associated POS is effectively managed with closure, often percutaneously, with marked and durable improvements in both gas exchange and quality of life. This supports the view that closure is curative in many cases. Moreover, anatomical and functional assessments are critical for tailoring treatment.

**Figure 4:**
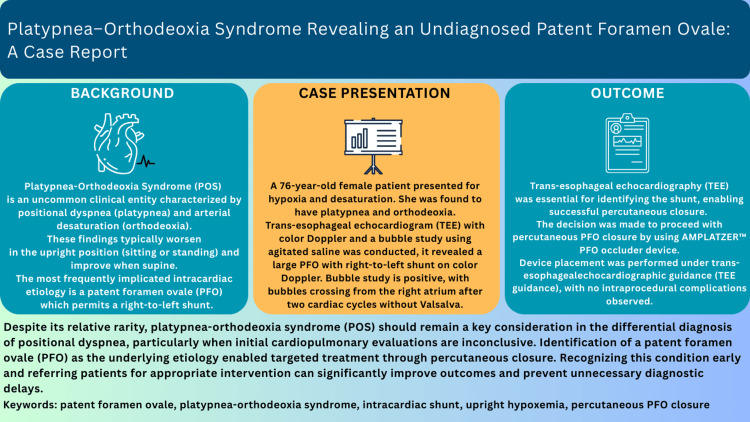
Educational summary figure. POS: platypnea-orthodeoxia syndrome; PFO: patent foramen ovale; TEE: transesophageal echocardiography; TTE: transthoracic echocardiography; RV: right ventricle.

Platypnea-orthodeoxia syndrome (POS) is a rare cause of positional dyspnea and hypoxemia, most commonly due to a patent foramen ovale causing right-to-left shunting. Early recognition allows effective targeted treatment (Figure 4) [6,11].

## Conclusions

Despite its relative rarity, platypnea-orthodeoxia syndrome (POS) should remain a key consideration in the differential diagnosis of positional dyspnea, particularly when initial cardiopulmonary evaluations are inconclusive and when there is a history of paradoxical embolic events. Physicians should consider POS in patients with unexplained dyspnea. Patent foramen ovale (PFO) became an important clinical condition to rule out in certain settings, particularly in young patients with cryptogenic stroke, although not confined only to this condition. In some cases, TTE may be inconclusive, and escalation to TEE is warranted. Identification of a PFO as the underlying etiology enables targeted treatment through percutaneous closure. Recognizing this condition early and referring patients for appropriate intervention can significantly improve outcomes and prevent unnecessary diagnostic delays.
